# An Atlas of Nomograms, Scoring Systems, and Predictive Tools to Guide Investigation or Management in Patients with Suspected or Confirmed Vesicoureteral Reflux: A Comprehensive Review of the Literature

**DOI:** 10.3390/jcm15010320

**Published:** 2026-01-01

**Authors:** Leo Edward FitzGerald Gradwell, Sanjeev Madaan, Bhaskar K. Somani

**Affiliations:** 1 University Hospital Southampton NHS Foundation Trust, Southampton SO16 6YD, UK; 2 Dartford & Gravesham Trust, Dartford DA2 8DA, UK

**Keywords:** vesicoureteral reflux, UTI, risk stratification, continuous antibiotic prophylaxis, machine learning

## Abstract

Background: Vesicoureteral reflux (VUR) contributes significantly to recurrent childhood urinary tract infections and renal scarring, yet predicting which patients will develop adverse outcomes or benefit from specific investigations or treatments remains challenging. Numerous prognostic tools have been proposed, but none have achieved widespread adoption. Methods: A comprehensive search of the literature available on MEDLINE, PUBMED, Embase, Emcare, CINAHL, and Google Scholar was performed to identify combinations of factors, scoring systems, ratios, models, and tools relating to VUR. This included predicting the spontaneous resolution of established vesicoureteral reflux, the risk of breakthrough urinary tract infections (UTIs), and guiding clinical decision making regarding the need for VCUG in patients with UTIs, continuous antibiotic prophylaxis (CAP), or surgical intervention in patients with confirmed VUR. Articles were included if they either described or validated a predictive tool that was designed to aid clinical decision making in patients with either suspected or confirmed VUR with regards to investigation or management strategies. All the studies included were then analysed, and the predictive tools have been summarised in a narrative format. Results: Seventeen predictive tools developed over thirty-nine years were identified: six predicting spontaneous resolution, four predicting breakthrough urinary tract infection (BTUTI) on CAP, two determining which children benefit from CAP, and five estimating the probability of VUR or high-grade VUR after a first febrile UTI. Approaches ranged from radiological ratios to multifactorial clinical–radiological scores and machine-learning models. Only five tools had any external validation, and none demonstrated sufficient reliability for universal clinical use. Significant heterogeneity in design, imaging interpretation, inclusion criteria, and outcome definitions limited comparison and wider applicability. Conclusions: This atlas provides the first consolidated overview of prognostic tools in paediatric VUR. Future development should prioritise multicentre, prospectively validated models that integrate established clinical and radiological predictors with transparent computational methods to create practical, generalisable risk-stratification frameworks for routine care.

## 1. Introduction

Vesicoureteral reflux (VUR) is a leading cause of recurrent urinary tract infection in children, with approximately 1–2% of the paediatric population being affected [1], and has a well-established association with the development of renal scarring [2]. Although it is postulated that approximately one in three infants presenting with urinary tract infections may have a degree of VUR [3], not all of these will go on to develop long-term kidney damage [4,5]. Conventional management of children suspected of having vesicoureteral reflux has included using video cystourethrogram (VCUG) scans to confirm the diagnosis and management with continuous antibiotic prophylaxis or surgical intervention [6,7]. However, concerns regarding exposure to potentially unnecessary radiation doses and the development of antimicrobial resistance have called traditional diagnostic and therapeutic strategies into question in recent years [8,9].

It remains an ongoing challenge for clinicians to determine which children with VUR will experience spontaneous resolution, recurrent urinary tract infection, renal scarring, and, potentially, complications of resultant chronic kidney disease [10,11,12,13,14,15]. Multiple publications have appeared in the literature over the past 40 years that describe predictive methods, models, scores, rules, nomograms, and ratios (referred to collectively hereafter as ‘predictive tools’) to stratify which children should undergo a VCUG for suspected VUR and/or guide management strategies for those with a confirmed diagnosis. Despite the variety of options, many of the tools remain relatively unknown within the field, which, alongside the relative rarity of the condition, may contribute to why they remain unvalidated and why no tool has thus far achieved universal adoption. This atlas is intended to serve as an easily digestible up-to-date summary of the current spectrum of predictive tools available for use and to provide a narrative appraisal of each to facilitate clinicians and researchers to easily find and evaluate the current tools.

## 2. Methods

A comprehensive search of the literature available on MEDLINE, PUBMED, Embase, Emcare, CINAHL, and Google Scholar was performed to identify combinations of factors, scoring systems, ratios, models, and tools relating to predicting the spontaneous resolution of established vesicoureteral reflux, the risk of breakthrough urinary tract infections, and guiding clinical decision making regarding the need for VCUG in patients with urinary tract infections, continuous antibiotic prophylaxis, or surgical intervention in patients with confirmed VUR. Search terms, including Boolean operators, included “vesicoureteral reflux” OR “VUR” AND “prognosis” OR “predict” OR “ratio” OR “model” OR “score” OR “scoring system” OR “nomogram” to identify suitable articles. All the articles including paediatric patients (under 18 years of age) were considered for inclusion. A total of 1706 articles were retrieved, and, after duplicates were removed, 327 abstracts were screened. Articles were included if they either described or validated a predictive tool that was designed to aid clinical decision making in patients with either suspected or confirmed vesicoureteral reflux with regards to investigation or management strategies. Articles were excluded if they did not describe or validate a specific predictive tool consisting of at least two measurable factors that can be used in combination to predict clinical outcomes in paediatric patients with possible or confirmed vesicoureteral reflux. All the studies included were then analysed, and the predictive tools have been summarised in a narrative format, including details about external validation and the citation count of the original published article on Google Scholar.

This work is intended as an atlas-style narrative review rather than a PRISMA-compliant systematic review. We used a structured search to identify candidate predictive tools, but we did not perform formal systematic review procedures (e.g., PRISMA flow reporting, duplicate screening by independent reviewers, or a risk-of-bias assessment).

Validation terminology: Internal validation refers to testing model performance using resampling (e.g., bootstrapping), split-sample approaches, or cross-validation within the derivation dataset; external validation refers to evaluation in an independent cohort from a different setting and/or time period. Where cross-validation is reported, the type is stated when available.

## 3. Results

In total, we have found and presented 17 distinct predictive tools that have been described over a span of 39 years. Six predict the spontaneous resolution of confirmed reflux, four predict breakthrough urinary tract infections whilst being treated with continuous antibiotic prophylaxis, two are designed to guide clinicians in deciding which patients to prescribe continuous antibiotic prophylaxis to, and five predict which patients presenting with a first febrile urinary tract infection should undergo VCUG to investigate possible VUR. One tool is based purely on clinical/serological factors, two are based purely on radiological findings, twelve utilise a combination of clinical, serological, and radiological factors that are inputted by the clinician and calculated using a formula, and four utilise computational or machine-learning methods. Several tools express their outcomes as percentages of risk, whilst others stratify patients into risk categories. Only five out of the seventeen predictive tools have, to date, undergone some form of external validation.

The section below provides a structured narrative summary of each tool (derivation variables, intended use, and reported validation), followed by a brief appraisal of practical considerations; broader synthesis is provided in the discussion.

For the purposes of this atlas, the following terminology applies:

- BBD = Bladder and bowel dysfunction, defined as issues relating to bladder and bowel functions.
- VCUG = Voiding cystourethrogram.
- CAP = Continuous antibiotic prophylaxis, defined as a regular low dose of prophylactic antibiotics to prevent infection.
- Spontaneous resolution = The spontaneous resolution of vesicoureteral reflux, defined as the complete resolution of previously confirmed reflux on repeat VCUGs.
- BTUTI = Breakthrough urinary tract infection, defined as a urinary tract infection that occurred whilst on continuous antibiotic prophylaxis therapy.
- Predictive tool(s) = Predictive method(s), specific combinations of individual factors, models, scores, rules, nomograms, risk classification systems, and ratios, which purpose is to aid clinical decision making through quantifying possible outcomes.

Note on terminology: Some older studies use ‘dysfunctional elimination syndrome (DES)’, typically referring to a combination of lower urinary tract dysfunction and constipation. In this atlas, we use BBD as the umbrella term, and where DES is used in the original paper, we report it as described and interpret it as BBD ± constipation unless otherwise specified.

## 4. Predictors of Spontaneous Resolution (Summarised in Table 1)

### 4.1. The Ureteral Diameter Ratio—Hellström et al. (1986) [16]

The ureteral diameter ratio (UDR) is a simple ratio first described by Hellström et al. (1986) [16] in the context of children diagnosed with low-risk vesicoureteral reflux. The ratio is calculated by measuring the largest ureteral diameter within the false pelvis (defined as the area below the most superior aspect of the ilia) in millimetres and dividing this by the distance from the bottom of the L1 vertebral body to the top of the L3 vertebral body in millimetres, as seen on a voiding cystourethrogram [16].

This foundational work was then built upon by Cooper et al. (2012) [17], who performed a retrospective review of 79 patients treated for VUR in a single institution between 1988 and 2004. After calculating the UDR for the patients, based on their initial VCUG, and reviewing the notes for future resolution or surgical correction, they determined that both a high VUR grade and a larger UDR were positively correlated with the persistence of the reflux and the need for surgical intervention. Cooper et al. (2012) noted that UDR outperformed the reflux grade as a predictor of clinical outcomes but did not establish any numerical cutoff points for UDR to be used as a predictor of specific outcomes [17].

A cutoff value of 0.43 was suggested by Arlen et al. (2017) [18] after analysing the data from 147 children and using multivariate analysis to look for predictive factors for the early spontaneous resolution of the reflux. UDR was found to correlate significantly with spontaneous resolution, with a *p*-value of <0.0001. No child in the cohort with a UDR of 0.43 or above experienced spontaneous resolution, and only three (4.5%) of the patients who experienced resolution had a UDR of >0.35 [18]. In a separate publication, Arlen et al. (2017) also demonstrated with statistical significance that increasing UDR correlated with an increased risk of breakthrough urinary tract infections after the analysis of data from 150 children [19].

Wong et al. (2023) [20] further validated the prognostic utility of UDR in predicting the spontaneous resolution of the reflux after the analysis of 101 patient records and proposed that a cutoff value of 0.26 be used to delineate patients into a high risk of persistence (>0.26) and a low risk of persistence (<0.26) [20]. Krishnan et al. (2024) [21] performed a systematic review and a meta-analysis that concluded that UDR is correlated with the spontaneous resolution of the reflux, breakthrough urinary tract infections, persistence (even after endoscopic treatment), and the need for surgical intervention; however, they concluded that optimal cutoff values could not be determined from the available literature [21].

The ureteral diameter ratio is, therefore, a simple and easily utilised tool that requires only the interpretation of a VCUG to give validated prognostic information regarding several important clinical outcomes in patients with VUR. It has been externally validated in multiple independent research articles, with consistent results; however, to date, no cutoff values have been agreed to across the literature. As well as being a useful standalone prognostic tool, it has been incorporated into other models and has demonstrated that it has improved those models’ predictive accuracies [22,23]. The lack of established cutoff values and validation in prospective studies remains a barrier to the UDR being used widely as a prognostic tool, and these issues need to be addressed for it to be used universally.

**Table 1:** Predictors of spontaneous resolution of vesicoureteral reflux.

Summary Table of Predictive Tools						
Author + Year	Name of Prognosticator/Tool	Variables Included/Description	Pros	Cons	Validation	Citation Count
Hellström et al., 1986 [16]	Ureteral diameter ratio	Variables included: Largest ureteral diameter within the false pelvis (in millimetres) Distance from the bottom of the L1 vertebral body to the top of the L3 vertebral body (in millimetres) The ratio of these two measurements = Ureteral Diameter Ratio (UDR) A simple, radiographic ratio first described in 1986 to assess vesicoureteral reflux (VUR) severity in children Calculated using measurements from a voiding cystourethrogram (VCUG). Initially applied to children with low-risk VUR; later validated as a predictor for spontaneous resolution, persistence, and surgical intervention in VUR	Simple to calculate using standard VCUG imaging Objectively quantifiable and reproducible Shown to outperform reflux grade alone as a predictor of clinical outcomes Demonstrated correlations with multiple clinically relevant outcomes: Spontaneous resolution of VUR Persistence or the need for surgery Breakthrough urinary tract infections (UTIs) Has improved predictive accuracy when incorporated into other prognostic models	No universally agreed numerical cutoff values across studies Most studies are retrospective; limited prospective validation Requires precise imaging measurements that may vary between institutions	External	55
Kirsch et al., 2014 [24]	Vesicoureteral Reflux Index	Variables included: Sex: Female = 1 point Ureteral anomalies: Complete duplication or periureteral diverticulum (PUD) = 1 point VUR grade: Grades 4–5 = 1 point Reflux timing during VCUG: Voiding only = 1 point Late filling = 2 points Mid-filling = 3 points Total possible score: 0–6 points Lower scores → Higher likelihood of spontaneous resolution A numerical scoring system developed to predict the probability of the spontaneous resolution of vesicoureteral reflux (VUR) in children under 2 years of age Provides a percentage-based estimate of the resolution probability over 1–3 years, based on the total score	Simple Reproducible Validated in both children under 2 years and older Simple and easy-to-use numerical system Quantifies risk, allowing for clear patient counselling Can predict spontaneous resolution and breakthrough urinary tract infections (UTIs) Shown to outperform VUR grade and UDR in predicting breakthrough UTIs	Predominantly validated in children under 2 years old—limited generalisability to older patients Dependent on VCUG findings, which are subject to interobserver variability and institutional imaging differences No prospective validation has yet been conducted Predictive accuracy decreases with higher index scores and longer time since the diagnosis	External	63
Estrada et al., 2009 [25]	Nomograms	Variables included: Age at presentation Sex Grade of reflux Laterality: Unilateral vs. bilateral reflux Ureteral anatomy: Single vs. duplex ureter Mode of clinical presentation: e.g., postnatal evaluation for prenatal hydronephrosis or sibling screening Derived from a retrospective analysis of 2462 children with vesicoureteral reflux (VUR) treated between 1998 and 2006 at a single high-volume centre Predictive nomograms were developed to estimate the time to VUR resolution. Each nomogram corresponds to a specific combination of predictive factors, providing a cumulative probability of reflux resolution at 1–5 years, expressed as a percentage.	Offers an individualised prediction for each patient, using specific combinations of variables Provides an easy-to-use, visual tool that clinicians can apply during counselling Outputs are expressed as percentages, which are easily interpretable and useful for patient and family discussions.	May be less applicable in atypical presentations Derived from retrospective, single-centre data, which may introduce bias Requires accurate input for all the variables; missing data may reduce utility. May be less adaptable to centres with differing patient populations or management protocols	None	205
Sjöström et al., 2020 [26]	Scoring system	Variables included: Sex: Male = 0 points; Female = 4 points Breakthrough urinary tract infections (UTIs): No = 0; Yes = 3 Renal damage (by DMSA or MAG3 scans): None = 0; Focal = 2; Generalised = 4 Glomerular filtration rate (GFR): Normal = 0; Subnormal = 3 Total score range: 0–14 points A point-based scoring system developed to predict the likelihood of downgrading vesicoureteral reflux (VUR) from grade 4/5 to grade 2 or below. Derived from a prospective cohort study of 89 infants (median age: 2.5 months) with grade 4/5 VUR, followed up to 39 months Risk variables were collected at 12 months of age. Scores 0–4: High probability of VUR ≤ grade 2 at the final follow-up Scores 4–8: Intermediate probability Scores 8–14: Low probability of VUR ≤ grade 2 at the final follow-up An attempted model for predicting absolute VUR resolution could not be generated due to event rarity.	Simple and numerical, facilitating easy interpretation Based on prospective data collection, unlike many other models Can be used for early risk stratification and family counselling Internally validated using bootstrapping (5000 resamples), supporting model stability	Only demonstrated in infants with high-grade VUR currently Not all parameters may be readily available across diverse clinical settings Small cohort size (n = 89) limits statistical power and generalisability Requires multiple investigations (VCUG, DMSA/MAG3 scans, and GFR testing), which may not be routinely available Predicts the downgrading of reflux, not complete resolution, making direct comparison with other tools challenging	Internal	5
Knudson et al., 2007 [27]	Computational model	Variables included: Reflux grade Age at diagnosis Bladder volume at reflux onset History of prenatal hydronephrosis Renal scan data (included in Nepple et al., 2008 [28] update) Renal scarring or decreased relative renal function (≤40% in refluxing kidney) A computational predictive model developed using retrospective data from 205 children with primary VUR Multiple modelling approaches tested; a linear support vector machine was selected initially for the best predictive accuracy. VUR resolution or persistence at 1 and 2 years post diagnosis Updated model by Nepple et al. (2008) [28] with enhanced predictive variables	Provides individualised predictions using patient-specific data Demonstrated high predictive accuracy Accessible online, facilitating clinical application without manual calculation	Evaluation has demonstrated inter-calculator variability Less accurate in older children Highly dependent on data quality Requires advanced diagnostics: bladder volume and renal scan data, which may not be universally available Developed primarily using retrospective data; prospective validation is lacking. Complexity may limit use in low-resource settings.	External	65
Tafazoli et al., 2025 [29]	Machine-learning model	Variables included: Renal scarring → predicts post-treatment febrile UTIs/renal scarring Bladder dysfunction → predicts post-treatment VUR persistence Developed to predict breakthrough UTIs, renal scarring, and the persistence of VUR in children treated with continuous antibiotic prophylaxis (CAP) Retrospective cohort of 225 children <2 years: 115 CAP patients for model development; 110 endoscopic injection patients as a comparator Random forest selected as final model; trained on 75% of CAP data, internally validated on 25% Predictive accuracy: 72–75% for VUR persistence/resolution and breakthrough UTI/renal scarring	Individualised risk stratification Data-driven decision support Simple to apply, as the final model uses only two significant clinical variables. High accuracy for the outcomes studied	Requires radiological assessments for variable acquisition, which may not be universally available The machine-learning infrastructure may be a barrier in low-resource settings. Does not address mild cases managed by observation Developed from retrospective data; internal validation only Reliance on variables that may be inconsistently reported	Internal	2
Acronym Key						
Acronym AUC BBD CAP CRP DMSA *E. coli* GFR MAG3 NNT PUD ROC RIVUR UDR UTD-P3 UTI VCUG VUR VURx/VUR Index WBC	Full Term Area Under the Curve Bladder and Bowel Dysfunction Continuous Antibiotic Prophylaxis C-Reactive Protein Dimercaptosuccinic Acid (scan) Escherichia coli Glomerular Filtration Rate Mercaptoacetyltriglycine (scan) Number Needed to Treat Periureteral Diverticulum Receiver Operating Characteristic (curve) Randomised Intervention for Vesicoureteral Reflux (trial) Ureteral Diameter Ratio Urinary Tract Dilation—Postnatal Grade 3 Urinary Tract Infection Voiding Cystourethrogram Vesicoureteral Reflux Vesicoureteral Reflux Index White Blood Cell (count)		Description/Context A measure of model accuracy or discrimination (from ROC analysis). A functional disorder affecting bladder and bowel control. A preventive long-term antibiotic therapy for recurrent urinary tract infections. A serum marker for inflammation, often used to detect/monitor infections. A nuclear medicine scan used to assess renal cortical scarring or function. A common bacterial cause of urinary tract infections. A measure of kidney function. A renal scintigraphy used to assess renal drainage and function. The number of patients who need to receive a treatment to prevent one additional bad outcome. A congenital or acquired outpouching near the ureter. A graphical plot used to assess the diagnostic accuracy. A multicentre clinical trial evaluating antibiotic prophylaxis in cases of vesicoureteral reflux. The radiographic ratio used to quantify ureteral dilation in vesicoureteral reflux. The highest grade of postnatal urinary tract dilation classification (severe hydronephrosis). An infection involving the urinary system (bladder, ureters, and kidneys). An imaging study used to diagnose and grade vesicoureteral reflux. The backflow of urine from the bladder to the ureters/kidneys. A composite scoring tool for predicting reflux resolution in children. A laboratory measure of immune cell levels, often elevated in infection.			

### 4.2. The Vesicoureteral Reflux Index—Kirsch et al. (2014) [24]

The vesicoureteral reflux index (VURx) is a numerical scoring system that was developed by Kirsch et al. (2014) [24] to predict the chance of spontaneous resolution in children under 2 years of age who have been diagnosed with vesicoureteral reflux. The index was derived after a retrospective analysis of all the children who received a diagnosis of VUR whilst under 2 years of age, over a 5-year period between 1 January 2006 and 31 December 2010 from a single-centre database. A multivariate analysis demonstrated that the female sex, high reflux grades (grades 4–5), ureteral anomalies (e.g., duplication or periureteral diverticula), and reflux timing during a voiding cystourethrogram were all independent predictors of the delayed resolution or persistence of the reflux [24].

By assigning each of the variables a numerical score: female sex = 1 point; the presence of a complete duplicate or PUD = 1 point; VUR grades 4–5 = 1 point; and reflux timing: voiding only = 1 point, late filling = 2 points, and mid-filling = 3 points, the total point score available is, therefore, 6 points, with lower scores indicating a greater probability of the spontaneous resolution of the reflux. The probability of resolution over 1–3 years can be expressed as a percentage based on the numerical scoring [24].

The vesicoureteral reflux index has been externally validated through several follow-up studies, including that by Arlen et al. (2016), who applied the index to retrospective data from 369 children under 2 years old from two institutions and demonstrated similar resolution rates as those in the original study [30]. Garcia-Roig et al. (2017) [31] evaluated the index in patients under 18 years of age, diagnosed with VUR after 2 years old, and confirmed that it can be used to predict spontaneous resolution/improvement in that age group; however, they noted that improvement/resolution appears less likely as both the index score and time from the diagnosis increase [31]. Arlen et al. (2020) subsequently performed a comparative analysis and demonstrated that the vesicoureteral reflux index outperformed both the VUR grade and ureteral diameter ratio in predicting breakthrough urinary tract infections, indicating that the index may have utility in this area as well [32].

In summary, the vesicoureteral reflux index is a simple scoring system that can be easily applied once calculated and provides a reliable numerical prediction of spontaneous resolution and, potentially, breakthrough infections and can be useful in counselling patients and their families. Although the index has been evaluated across a range of age ranges, it has been predominantly validated in children under 2 years of age, and its reliance on VCUG findings introduces a risk of interobserver heterogeneity in interpretation and differences in imaging techniques, therefore limiting its generalisability. It has also not been prospectively validated, and this will be required to make it universally applicable.

### 4.3. Nomograms—Estrada et al. (2009) [25]

Estrada et al. (2009) [25] described the derivation of several nomograms that can be used to predict the spontaneous resolution of vesicoureteral reflux, after a retrospective analysis of 2462 children referred to a single high-volume unit between 1998 and 2006. The authors identified the following variables as being independently predictive of VUR resolution through univariate and multivariate analyses: the age at presentation, sex, grade of the reflux, laterality (unilateral vs. bilateral), ureteral anatomy (single vs. duplex ureter), and mode of clinical presentation (e.g., postnatal evaluation for prenatal hydronephrosis or sibling screening). Cox proportional hazard regression was then performed to model the time to VUR resolution, and nomograms were developed using every possible combination of the identified predictive factors. The described nomograms can be used to provide a cumulative probability of the resolution of the reflux at annual intervals from 1 to 5 years, expressed as a percentage of the presenting cases.

Although no external validation has been published for these nomograms, which limits their reliability and generalisability, they offer distinct advantages as decision making and counselling aids for paediatric urologists. The fact that a unique nomogram has been developed for each combination of predictive factors allows for a relatively individualised approach for each patient. The nomograms also offer an easy-to-use tool, and the expression of probability as a percentage is easily understood and explained to patients and their families, which means that these have the potential to be useful clinical tools to aid decision making and patient counselling [25].

### 4.4. The Scoring System—Sjöström et al. (2020) [26]

The Sjöström scoring system was originally published in 2020 [26]. It is a point-based scoring system, with a score range between 0 and 14 points available and derived from the following four variables: sex (male = 0 and female = 4), the presence of breakthrough urinary tract infections (no = 0 and yes = 3), the presence of renal damage (none = 0, focal = 2, and generalised = 4), and the glomerular filtration rate (normal = 0 and subnormal = 3). In the study, renal damage was assessed through DSMA or MAG3 scans [26].

The score can be used to predict the likelihood of downgrading VUR from grade 4/5 to grade 2 or below. The variables included in the scoring system were identified through a prospective analysis of 89 infants with VUR grade 4 or 5, diagnosed at a median age of 2.5 months and followed to 39 months; the risk variables were collected at 12 months of age. Univariate analysis and, subsequently, multivariate analysis were used to identify factors that were independently predictive of the described outcome and incorporated into the above score.

Sjöström et al. (2020) [26] defined those with a score of 0–4 as having a high probability of VUR being less than or equal to 2 at the final follow-up, scores between 4 and 8 as having an intermediate probability of VUR being less than or equal to 2, and scores between 8 and 14 being classed as having a low probability of VUR being less than or equal to 2 at the final follow-up. The authors attempted to generate a score to predict the absolute resolution of VUR; however, due to the rarity of this, even in their cohort, they could not do so. The scoring system was internally validated using a bootstrapping technique using 5000 samples drawn with replacement from the original population, sampling 5000 studies of the same size as that of the original sample (*n* = 89) [26]. No external validation has been published to date.

The Sjöström scoring system provides a simple numerical scoring system using information gathered early in life to aid in clinical decision making and patient/family counselling. However, due to the requirement for multiple different imaging studies to be performed, as well as blood tests, and the relatively small sample size, coupled with a lack of external validation studies, it is not currently able to be used universally in clinical practice.

### 4.5. The Computational Model—Knudson et al. (2007) [27]

Knudson et al. (2007) [27] built a computational model based on a retrospective analysis of 205 children with primary vesicoureteral reflux treated at a single institution in Iowa between 1988 and 2004. Clinical data extracted included the age, gender, presenting symptom, reflux grade, laterality, whether reflux occurred during filling or voiding, initial bladder volume at the onset of the reflux, and complete ureteral duplication. Using spontaneous resolution and unresolved reflux (if the patient underwent surgery or had persistent reflux on follow-up cystograms 1 and 2 years after the diagnosis) as designated outcomes, the team set out to create a computational model to predict the above outcomes. Two datasets were created and randomly assigned to a modelling set of 155 patients for training and a cross-validation set of 50 patients for internal validation. Multiple computational models were built using neUROn++ and C++ programs to optimise the predictive output. A linear support vector machine was chosen due to having the best predictive accuracy. The final model utilises the reflux grade, age at diagnosis, bladder volume at the reflux onset, and history of prenatal hydronephrosis and modelled outcomes for 1 and 2 years after the diagnosis. The ROC curve areas of the final model were 0.819 and 0.86 for the 1- and 2-year models respectively. The model was inputted into JavaScript to enable clinicians to easily input patient-specific data and obtain individualised predictions through an online platform [27].

A re-evaluation of the initial model was described by the team that developed the initial model in Nepple et al. (2008) [28]. In this iteration, the team included renal scan data on renal scarring or decreased relative renal function (40% or less in the refluxing kidney) and tested the new model on data from 161 children. The datasets were randomly assigned to either a modelling set (111 children) or a cross-validation set (50 children). A linear regression model was selected as the superior predictive model both in this publication and when compared to the previous model, with an ROC area of 0.945 for predicting reflux resolution in the 2-year model [28].

The modified model was externally validated by Shiraishi et al. (2009), using a retrospective cohort of 82 Japanese children, and the team found that the model predicted resolution by 2 years post diagnosis, with an overall accuracy of 80.5%, a sensitivity of 82.5%, a specificity of 78.6%, a positive predictive value of 78.6%, and a negative predictive value of 82.5% [33].

In summary, the Knudson/Nepple computational model seems to be a potentially useful model; however, further prospective external validation will be required to ensure its reliability. Being available through the internet makes it openly accessible; however, it does rely on knowledge of the bladder and renal status obtained through advanced tests that may not be universally available.

### 4.6. The Machine-Learning Model—Tafazoli et al. (2025) [29]

Tafazoli et al. (2025) [29] developed a machine-learning model for predicting several clinical outcomes in children with confirmed vesicoureteral reflux who are being treated with continuous antibiotic prophylaxis (CAP). The outcomes defined in the original study include breakthrough urinary tract infections, renal scarring, and the persistence of the reflux.

They performed a retrospective analysis of data for two hundred twenty-five children under 2 years of age, taken from two separate units: one hundred fifteen children treated with continuous antibiotic prophylaxis at a paediatric nephrology clinic were used for model development, and one hundred ten children who were treated at a paediatric surgery unit with endoscopic injections of a dextranomer/hyaluronic acid copolymer were used as a comparator group. The data gathered included the sex, age at diagnosis, medications, VUR laterality, dimercaptosuccinic acid (DMSA) differential renal function, VUR grade, dilating or non-dilating reflux in ultrasonography, and the presence of febrile UTI, prenatal hydronephrosis, ureteral anomaly, bladder dysfunction, neuropathic bladder, failure to thrive, and renal scarring [29].

After multivariate analysis, it was demonstrated that only renal scarring was significantly associated with post-treatment febrile UTIs and/or renal scarring (*p*-value: 0.007), and bladder dysfunction was the only factor significantly associated with post-treatment VUR persistence (*p*-value: 0.004) [29]. Thus, these were used for the model derivation.

Five models of a logistic regression, a random forest, an SVM, gradient boosting, and a neural network were developed for both outcomes (breakthrough febrile UTI/renal scarring and persistence/resolution of the reflux) and the random forest was the best model for both outcomes. The final model was trained using 75% of the data from the CAP group, and 25% was used for internal validation. The final model reported an overall predictive accuracy of between 72% and 75% for VUR persistence/resolution and breakthrough UTI/renal scarring, respectively [29].

This machine-learning model serves as a potentially useful tool to discriminate between children who can be trialled on continuous antibiotic prophylaxis versus those who should be considered for a surgical intervention. The fact that it utilises only two clinical variables makes it relatively simple to apply; however, the acquisition of these variables does require the interpretation of radiological assessments that may not be universally available. Machine-learning technology is also potentially a limiting factor in different economic and technological environments, and the model does not facilitate any decision making that does not involve active treatment, which precludes its use in mild cases, where observation might be considered.

Prospective external validation in larger cohorts would be necessary to demonstrate widespread applicability and, alongside the reliance on variables that may be inconsistently reported, represents the greatest limitation of this model at this stage.

For computational and machine-learning tools, we first summarise methods and report performance, as stated in the original manuscript; any additional commentary on practicality or limitations reflects our narrative appraisal and is synthesised in the discussion.

## 5. Predictors of Breakthrough Urinary Tract Infections (Summarised in Table 2)

### 5.1. The Risk Prediction Model—Dias et al. (2010) [34]

Dias et al. (2010) [34] developed a risk prediction model for the development of breakthrough urinary tract infections in paediatric patients with primary vesicoureteral reflux. The authors reviewed retrospective data derived from patients treated in a single tertiary renal unit in Brazil between 1970 and 2008. A total of 740 patients were identified and included. Binary logistic regression was used to identify independent predictors of recurrent UTIs, defined as more than one episode of febrile UTI during the follow-up [34].

The following variables were considered: race, gender, clinical presentation (foetal hydronephrosis/UTI), lower urinary tract dysfunction (absence/presence), constipation, dysfunctional elimination syndrome (combined lower urinary tract dysfunction and constipation), VUR grade (mild/moderate/severe), reflux laterality (unilateral/bilateral), renal damage (absence/unilateral/bilateral), and the severity of the renal damage (focal renal scar/multiple scars/contracted renal unit). Only five variables were found to be independently predictive of the defined outcome: UTI as the initial presenting problem, female sex, an age of < 6 months at presentation, the presence of dysfunctional elimination syndrome, and a reflux grade of 4–5 [34].

A numerical weighting was calculated for each of the independently predictive variables, and a total score for each patient was derived from summing the weightings present. The total score range, therefore, is between 0 and 9.05. The prognostic risk score was presented as: <4.25 = low risk, 4.25–5.05 = intermediate risk, and 5.05–9.05 = high risk. The overall accuracy of the scoring was found to be acceptable, with a C-statistic of 0.68 and a Hosmer–Lemeshow goodness-of-fit test *p*-value of 0.97. The calculated UTI incidence rates per 1000 person–months for each risk group were 4.3 (95% CI, 3.2, 5.6), 7.9 (95% CI, 6.7, 9.1), and 11.3 (95% CI, 9.9, 12.8) for the low-risk, intermediate-risk, and high-risk groups, respectively [34].

This risk-prediction model offers a simple and easily applied tool using patient information that is likely to be readily available in most contexts; however, to date, no external validation has been published, and thus it cannot be confidently said to apply outside of its derivation cohort. External prospective validation studies will be necessary to ensure its reliability across a range of patient populations.

### 5.2. The Risk Prediction Model—Hidas et al. (2015) [35]

Another risk prediction model developed to predict breakthrough urinary tract infections was published by Hidas et al. (2015) [35]. They performed a retrospective analysis of clinical and demographic data from 252 children with vesicoureteral reflux treated as a single unit between June 2008 and December 2010 to identify independent risk factors for breakthrough urinary tract infections. Variables were initially evaluated for associations with breakthrough UTIs, using Fisher’s exact tests, and those that demonstrated associations in the unadjusted bivariate analysis were included in an initial multivariate logistic regression model. Subgroup analysis was subsequently performed, comparing variable associations in patients with lower-grade VURs (grades 1–3) and those with higher-grade VURs (grades 4–5) [35].

The final model was based on the following factors deemed to be significant for prediction: sex, primary presentation as a urinary tract infection, grade 4–5 reflux, and the presence of bladder and bowel dysfunction. The individual factors were multiplied by their individual beta-coefficients (the natural log of the odds ratio) and then summed to give a total score. This score was evaluated based on the 2-year probability of a breakthrough UTI and presented as a percentage risk. The authors were then able to categorise patients based on their score as being at low, intermediate, or high risk of having a breakthrough UTI during that 2-year period [35].

The model demonstrated good overall accuracy, with areas under the ROC curves of 0.76 in the original derivation cohort and 0.8 in a prospective cohort of 56 children evaluated within the original study by way of validation [35].

Hidas et al. (2015) [35] used their model to develop a web-based scoring system that presented the overall risk of breakthrough UTIs as a percentage. The score is called the iReflux score and can be readily accessed online. Of note, the current iteration of the score includes details regarding the patient’s age, laterality of reflux, and circumcision status despite these not affecting the probability outcome. The inclusion of the circumcision status in the online calculator is addressed in the original publication by justifying that, like bladder and bowel dysfunction, the circumcision status has been linked in other studies to UTIs in children and is a modifiable factor.

To date, no external validation is available in the literature for this risk prediction score, which is the major limitation. Given that the score was derived from a relatively small cohort from a single centre; a prospective, large-cohort external validation will be required to ensure that the score can be used in widespread clinical practice. The stratification of patients into risk categories, expressions, or risks in the form of simple percentages and the inclusion of easily accessible clinical and demographic variables do make this an attractive option if it is demonstrated to be reliable through external validation.

### 5.3. The Prediction Model—Yang et al. (2025) [36]

Yang et al. (2025) [36] developed a risk prediction model and nomogram for breakthrough urinary tract infections in children being treated for primary vesicoureteral reflux after a retrospective analysis of 193 patients treated between January 2019 and August 2021 at a single specialist centre in China. Data were extracted from clinical records and VCUG scans and subjected to univariate and multivariate analyses to identify independent predictors, and the team developed a model, which they compared to two other established predictors: the urethral diameter ratio and the vesicoureteral index [36].

The following variables were considered for inclusion: the age at presentation, sex, number of UTIs before the VUR diagnosis (variable “UTI history”), and circumcision status. Relevant imaging parameters included the anteroposterior pelvis diameter, ureteral anomalies, VUR grade at diagnosis, VUR timing at cystography, bladder morphological changes, sacral ratios, ureteropelvic junction diameter of the ureter, ureterovesical junction diameter of the ureter, maximum ureteral diameter, ureteral tortuosity, distal ureteral diameter, and distal UDR. Multivariate logistic regression analysis revealed that the sex, high-grade VUR, and ureterovesical junction diameter were the only independent predictors of breakthrough UTIs (*p* < 0.05) and, thus, were used to construct the risk prediction model and nomogram [36].

The performance of the model was assessed for discrimination, calibration, and clinical benefit. The calibration curve was then used to compare between the observed and predicted outcomes. The receiver operating characteristic (ROC) curve was used to evaluate the model’s discriminative ability. The optimal threshold probability was based on the Youden index from the model’s ROC analysis. A decision curve analysis (DCA) was used to assess the clinical net benefit of the nomogram [36].

Yang et al. (2025) [36] performed an internal validation of the nomogram using 1000 bootstrap sample corrections. The optimism-corrected concordance index (C-index) was 0.73 after corrections, with a calibration slope of 0.93, and the areas under the curves for Yang et al.’s model, the UDR, and the VURx in predicting the occurrence of breakthrough infections were 0.736, 0.680, and 0.546, respectively [36].

This prediction model and nomogram show promise for being a simple, user-friendly tool that can be applied easily in a clinical setting. The clinical and radiological variables included are generally easily available, and the simplicity in interpretation makes it an attractive option. The primary limitation of the model’s use at present is the lack of any external validation, limiting its generalisability. Future prospective studies with larger populations across a diverse background are necessary to bring this model to mainstream practice.

### 5.4. The Computational Model—Troesch et al. (2021) [37]

Troesch et al. described their development of a computational model to predict early-breakthrough urinary tract infections in children with vesicoureteral reflux. Retrospective records were reviewed from 864 children treated for primary vesicoureteral reflux in a single centre in Iowa between 1988 and 2018, with the intention of developing a model to predict breakthrough urinary tract infections. Records were screened for the VUR grade, laterality of the reflux, VUR during filling or voiding, initial bladder volume at the onset of the VUR, ureteral duplication, voiding dysfunction, distal ureteral diameter ratio, and number of UTIs prior to the VUR diagnosis. A total of 136 children were found to have all the data available. Using logistic regression and multiple neural network architectures through neUROn++ and C++ programs, multiple predictive models utilising a range of variables were developed and assessed. The best-performing computational model was one that utilised all the variables and was demonstrated to have an area under the curve of 0.802 [37].

This computational model demonstrates high predictive accuracy, which shows that if externally validated in diverse prospective cohorts, it may prove to be a useful tool in clinical practice. The main limitations of it are that the authors limited the outcome duration to breakthrough UTIs within 13 months of the VUR diagnosis due to concerns about the relevance of clinical data changing through development, thereby limiting its current usefulness to predicting UTIs in the first 13 months. It should also be noted that the model’s reliance on a large amount of clinical data and access to advanced computational technology disadvantages it when compared to some of the other predictive tools available.

**Table 2:** VUR predictors of breakthrough infections.

VUR Predictors of Breakthrough Infections						
Author + Year	Name of Prognosticator/Tool	Variables Included/Description	Pros	Cons	Validation	Citation Count
Dias et al., 2010 [34]	Risk prediction model	Variables included: Sex Primary presentation as a urinary tract infection VUR grade 4–5 Presence of bladder and bowel dysfunction Additional variables included in the online calculator: age, reflux laterality, and circumcision status; these do not alter probability outcomes but are included for context. Developed to predict breakthrough urinary tract infections (UTIs) in children with VUR Retrospective cohort study of 252 children from a single centre (2008–2010) Presented as a percentage probability of breakthrough UTIs over 2 years Allows stratification into low, intermediate, or high-risk categories Implemented as a web-based scoring system for ease of clinical use	Uses clinically accessible variables, facilitating easy application Demonstrated good predictive accuracy	Derived from a single-centre, relatively small cohort, limiting generalisability	None	95
Hidas et al., 2015 [35]	Risk prediction model	Variables included: UTI as an initial presenting problem Female sex Age < 6 months at presentation Presence of dysfunctional elimination syndrome VUR grade 4–5 Total score range: 0–9.05 <4.25 → Low risk 4.25–5.05 → Intermediate risk 5.05–9.05 → High risk Developed to predict the risk of breakthrough urinary tract infections in children with primary VUR Derived from a retrospective analysis of 740 patients treated at a single tertiary renal unit in Brazil (1970–2008) Risk groups were associated with UTI incidence per 1000 person-months: Low risk: 4.3 Intermediate risk: 7.9 High risk: 11.3	Individualised risk stratification Easy to use in clinical practice Simple and easily applied using routinely available patient data Provides quantitative risk stratification (low/intermediate/high) Simple online tool presenting percentage risk and risk categories	Derived from a single-centre, retrospective cohort, which may introduce bias Some variables in the online calculator (age, laterality, and circumcision status) do not influence the predicted probability, which may cause confusion.	None	54
Yang et al., 2025 [36]	Prediction model + nomogram	Variables included: Sex VUR grade (specifically, high-grade VUR) Ureterovesical junction diameter Developed through a retrospective analysis of 193 paediatric patients treated for primary vesicoureteral reflux between January 2019 and August 2021 at a single specialist centre in China The model identifies independent predictors of breakthrough urinary tract infections (UTIs) and presents them in a nomogram format for clinical use. It was compared against two other established predictors: UDR and VURx.	Simple and user friendly; can be easily applied in clinical settings Utilises routinely available clinical and radiological variables Demonstrates good discrimination Calibration and decision curve analyses indicate strong predictive performance and potential clinical benefit.	Single-centre dataset limits generalisability Retrospective study design	Internal	0
Troesch et al., 2021 [37]	Computational model	Variables included: VUR grade Laterality of reflux VUR during filling or voiding Initial bladder volume at the onset of the VUR Ureteral duplication Voiding dysfunction Distal ureteral diameter ratio Number of UTIs prior to VUR diagnosis Developed using a retrospective review of 136 children with vesicoureteral reflux Multiple predictive models were created using logistic regression and neural network architectures (implemented through neUROn++ and C++). The best-performing computational model included all the variables, achieving an AUC of 0.802 for predicting early breakthrough UTIs.	Demonstrated high predictive accuracy Incorporates a wide range of clinical and imaging data, potentially enhancing prediction precision Illustrates the potential of machine-learning/neural network approaches in paediatric urology prediction models	Requires large and detailed datasets, which may not always be available in clinical practice Dependence on advanced computational infrastructure and technical expertise limits routine implementation. Limited accessibility to model details (the full article is unavailable).	None	19
Acronym Key						
Acronym AUC BBD CAP CRP DMSA *E. coli* GFR MAG3 NNT PUD ROC RIVUR UDR UTD-P3 UTI VCUG VUR VURx/VUR Index WBC	Full Term Area Under the Curve Bladder and Bowel Dysfunction Continuous Antibiotic Prophylaxis C-Reactive Protein Dimercaptosuccinic Acid (scan) Escherichia coli Glomerular Filtration Rate Mercaptoacetyltriglycine (scan) Number Needed to Treat Periureteral Diverticulum Receiver Operating Characteristic (curve) Randomised Intervention for Vesicoureteral Reflux (trial) Ureteral Diameter Ratio Urinary Tract Dilation—Postnatal Grade 3 Urinary Tract Infection Voiding Cystourethrogram Vesicoureteral Reflux Vesicoureteral Reflux Index White Blood Cell (count)		Description/Context A measure of model accuracy or discrimination (from ROC analysis). A functional disorder affecting bladder and bowel control. A preventive long-term antibiotic therapy for recurrent urinary tract infections. A serum marker for inflammation, often used to detect/monitor infections. A nuclear medicine scan used to assess renal cortical scarring or function. A common bacterial cause of urinary tract infections. A measure of kidney function. A renal scintigraphy used to assess renal drainage and function. The number of patients who need to receive a treatment to prevent one additional bad outcome. A congenital or acquired outpouching near the ureter. A graphical plot used to assess diagnostic accuracy. A multicentre clinical trial evaluating antibiotic prophylaxis in cases of vesicoureteral reflux. The radiographic ratio used to quantify ureteral dilation in vesicoureteral reflux. The highest grade of postnatal urinary tract dilation classification (severe hydronephrosis). Infection involving the urinary system (bladder, ureters, and kidneys). An imaging study used to diagnose and grade vesicoureteral reflux. The backflow of urine from the bladder to the ureters/kidneys. A composite scoring tool for predicting reflux resolution in children. A laboratory measure of immune cell levels, often elevated in infection.			

## 6. Predictors of Those Who Benefit from Continuous Antibiotic Prophylaxis (Summarised in Table 3)

### 6.1. The Risk Classification System—Wang et al. (2018) [38]

Wang et al. (2018) sought to identify which patients with vesicoureteral reflux were at the greatest risk of breakthrough infections by re-evaluating the data from the RIVUR trial [38]. The RIVUR trial was a multisite, randomised, placebo-controlled trial involving 607 children with vesicoureteral reflux to evaluate antimicrobial efficacy, renal scarring, and antimicrobial resistance [39].

After retrospectively re-evaluating the trial data from all 607 patients. Wang et al. (2018) [38] performed a multivariable analysis to determine the factors that were independent predictors of breakthrough urinary tract infections. They concluded that the VUR grade (high vs. low), presence of bladder and bowel dysfunction, history of urinary tract infection recurrence, and presence of renal scarring were all significant predictors and developed a risk classification model utilising these variables. The model stratifies patients into low risk (circumcised males or females with a grade 1–3 reflux AND no evidence of bladder and bowel dysfunction/constipation) and high risk (uncircumcised males with a VUR Grade of I-III ± BBD/Constipation OR females with a VUR Grade of I-III and BBD/Constipation OR females and males with a VUR Grade of IV ± BBD/Constipation). Wang et al. calculated that the number needed to treat for low-risk patients was 18 and for high-risk patients was 5, with regards to treatment with prophylactic antibiotics to prevent breakthrough urinary tract infections. All the outcomes reported had *p*-values of <0.05 [39].

This risk model provides insight into whether patients benefit from continuous antibiotic prophylaxis, which is a pertinent clinical question, considering concerns regarding antimicrobial resistance. It is easily applied in clinical practice, as the categorisation is binary; however, currently, it has not been externally validated and so cannot be reliably applied outside of its original cohort.

### 6.2. The Machine-Learning Model—Bertsimas et al. (2021) [40]

Another attempt to generate a predictive model through the re-evaluation of the RIVUR trial data was undertaken by Bertsimas et al. (2021) [40]. In this case, the investigating team used the following variables: the VUR grade, serum creatinine level, race, gender, prior UTI symptoms (fever and dysuria), and weight percentiles to develop a machine-learning model to predict which patients would benefit from continuous antibiotic prophylaxis [40].

Two models were constructed in parallel, using a randomly selected 80% of the trial data: one to predict the risk of recurrent urinary tract infection whilst being treated with antibiotic prophylaxis and one without treatment with antibiotics. Predicted probabilities of recurrent urinary tract infections were generated from each model, and continuous antibiotic prophylaxis was assigned at various cutoff points of recurrent urinary tract infection risk reduction to evaluate the continuous antibiotic prophylaxis’s effectiveness. The final prediction model of recurrent urinary tract infections (continuous antibiotic prophylaxis/placebo) achieved an area under the curve of 0.82, indicating high predictive accuracy [40].

**Table 3:** Predictors of those who benefit from continuous antibiotic prophylaxis.

Predictors of Those Who Benefit from Continuous Antibiotic Prophylaxis						
Author + Year	Name of Prognosticator/Tool	Variables Included/Description	Pros	Cons	Validation	Citation Count
Wang et al., 2018 [38]	Risk classification system	Variables included: VUR grade (high vs. low) Presence of bladder and bowel dysfunction (BBD) History of recurrent urinary tract infections Presence of renal scarring Developed through a reanalysis of data from the RIVUR trial Used multivariable analysis to identify independent predictors of breakthrough UTIs Based on these predictors, the model stratifies patients into two risk groups for breakthrough infection: Low-risk group: NNT = 18 High-risk group: NNT = 5	Based on a large, multicentre, randomised trial dataset (RIVUR trial), enhancing the reliability of the findings Clinically relevant—directly informs antibiotic prophylaxis decision making Incorporates both clinical and imaging predictors	Focused on statistical reanalysis rather than real-time clinical applicability No external validation	None	61
Bertsimas et al., 2021 [40]	Machine-learning model	Variables included: VUR grade Serum creatinine Race Gender Prior UTI symptoms (fever, dysuria) Weight percentile Developed using re-evaluated data from the RIVUR trial Two parallel machine-learning models were created using 80% of the dataset: One predicting the risk of recurrent UTI with continuous antibiotic prophylaxis (CAP) One predicting the risk of recurrent UTI without CAP Predicted probabilities from both models were compared to assess the benefit of CAP. A 10% risk reduction cutoff threshold optimised outcomes, suggesting that giving CAP to ~40% of the patients minimised recurrent UTI rates. In the test set (20% of the data), patients whose CAP assignment matched the model’s recommendation had significantly lower recurrent UTI incidence (7.5% vs. 19.4%, *p* = 0.037).	Demonstrated high predictive accuracy Enables individualised treatment decisions—optimises CAP use to those most likely to benefit Uses comprehensive clinical and demographic data Supported by validation on a held-out test dataset from the same trial	Relies on computational resources and machine-learning expertise Based on secondary analysis of RIVUR data (retrospective application)	Internal	32
Acronym Key						
Acronym AUC BBD CAP CRP DMSA *E. coli* GFR MAG3 NNT PUD ROC RIVUR UDR UTD-P3 UTI VCUG VUR VURx/VUR Index WBC	Full Term Area Under the Curve Bladder and Bowel Dysfunction Continuous Antibiotic Prophylaxis C-Reactive Protein Dimercaptosuccinic Acid (scan) Escherichia coli Glomerular Filtration Rate Mercaptoacetyltriglycine (scan) Number Needed to Treat Periureteral Diverticulum Receiver Operating Characteristic (curve) Randomised Intervention for Vesicoureteral Reflux (trial) Ureteral Diameter Ratio Urinary Tract Dilation—Postnatal Grade 3 Urinary Tract Infection Voiding Cystourethrogram Vesicoureteral Reflux Vesicoureteral Reflux Index White Blood Cell (count)		Description/Context A measure of model accuracy or discrimination (from the ROC analysis). A functional disorder affecting bladder and bowel control. A preventive long-term antibiotic therapy for recurrent urinary tract infections. A serum marker for inflammation, often used to detect/monitor infections. A nuclear medicine scan used to assess renal cortical scarring or function. A common bacterial cause of urinary tract infections. A measure of kidney function. A renal scintigraphy used to assess renal drainage and function. The number of patients who need to receive a treatment to prevent one additional bad outcome. A congenital or acquired outpouching near the ureter. A graphical plot used to assess diagnostic accuracy. A multicentre clinical trial evaluating antibiotic prophylaxis in cases of vesicoureteral reflux. The radiographic ratio used to quantify ureteral dilation in vesicoureteral reflux. The highest grade of postnatal urinary tract dilation classification (severe hydronephrosis). Infection involving the urinary system (bladder, ureters, and kidneys). An imaging study used to diagnose and grade vesicoureteral reflux. The backflow of urine from the bladder to the ureters/kidneys. A composite scoring tool for predicting reflux resolution in children. A laboratory measure of immune cell levels, often elevated in infection.			

By assigning a risk reduction cutoff threshold of 10% for recurrent urinary tract infections, it was found that the minimal recurrent urinary tract infection per population level was achieved by giving continuous antibiotic prophylaxis to 40% of the patients with vesicoureteral reflux instead of everyone. In a test set of approximately 20% of the trial data, 51 patients were assigned to have continuous antibiotic prophylaxis, consistent with the model’s recommendation (continuous antibiotic prophylaxis if the recurrent urinary tract infection risk reduction was >10%). The incidence of recurrent urinary tract infections in this group was significantly lower when compared to those whose continuous antibiotic prophylaxis assignment differed from the model’s suggestion (7.5% vs. 19.4%, *p* = 0.037) [40].

In summary, Bertsimas et al. (2021) [40] have created a machine-learning model that appears to be able to accurately differentiate which patients will achieve the greatest benefit from continuous antibiotic prophylaxis. Whilst this is an attractive option thus far, it has not been externally validated and cannot, therefore, be reliably applied across paediatric urology practice. This, combined with the technological limitations of making this model widely accessible, is preclusive to general use.

## 7. Predictors of Those Who Benefit from VCUG After a First Febrile UTI (Summarised in Table 4)

### 7.1. The Oostenbrink Multivariate Model—Oostenbrink et al. (2000) [41]

The Oostenbrink multivariate model was described after a retrospective analysis of 140 children who presented, at an age of <5 years old, to hospital with a first episode of febrile urinary tract infection between September 1993 and September 1996. Data were collected from three large hospitals in the Netherlands. The purpose of the model was to predict which children presenting with their first febrile urinary tract infection may have vesicoureteral reflux and, thus, should be investigated with a VCUG [41].

The team interrogated the patients’ records for all the clinical and investigative details and performed, first, univariate and, subsequently, multivariate analyses to identify possible predictors. Clinical details included symptoms of a urinary tract infection specified by the patient’s age, sex, and family history of uropathy or constipation. Laboratory details included the white cell count, erythrocyte sedimentation rate, C-reactive protein level, and urine culture result. Radiological details included the ultrasound scan of the urinary tract, VCUG (if available), and MAG-3 scan (if available). After using a stepwise approach to include variables and refine predictive models, they concluded from their analysis that the sex, age, family history of uropathology, serum C-reactive protein level, and ureteral dilation in an ultrasound scan were all independent predictors of VUR [41].

The final clinical prediction model achieved an area under the ROC curve of 0.78, and a risk prediction score was produced that consists of a summation of the points attracted for each of the positive findings. Oostenbrink et al. (2000) concluded that the risk score showed promise but required prospective validation before being applicable in clinical practice [41].

Several studies have proceeded to externally validate the Oostenbrink model. Leroy et al. (2006) [42] tested the model using a retrospective cohort of 149 children and concluded that although the sensitivity was high, the specificity was low, at 3% for VURs of all grades and 13% for VURs of grade 3 or higher [41]. Sánchez Bayle et al. (2008) concluded, after a review including 267 infants, that the score did not effectively predict VUR [43], and Venhola et al. (2010) concluded, after applying the model to 406 patients, that the sensitivity was 24% for identifying refluxes of grade 3 or higher [44]. The external validation, therefore, suggests that this tool is not widely applicable to clinical practice.

**Table 4:** Predictors of those who benefit from VCUG after a first febrile UTI.

Predictors of Those Who Benefit from VCUG After a First Febrile UTI						
Author + Year	Name of Prognosticator/Tool	Variables Included/Description	Pros	Cons	Validation	Citation Count
Oostenbrink et al., 2000 [41]	Oostenbrink Multivariate Model	Variables included: Sex Age Family history of uropathology Serum C-reactive protein (CRP) Ureteral dilation in an ultrasound Developed from a retrospective analysis of 140 children <5 years old presenting with their first febrile UTI at three hospitals in the Netherlands (1993–1996) A risk score was created based on the summation of points assigned for each positive predictor. The ROC curve showed AUC = 0.78, indicating moderate discrimination.	Combines clinical, laboratory, and imaging data for a multifactorial prediction Produces a simple point-based risk score that can be applied in clinical settings	The initial study was retrospective and based on a single country dataset. External validation studies showed poor specificity and inconsistent predictive performances.	External	87
Leroy et al., 2012 [45]	Leroy Clinical Decision Rule	Variables included: Serum procalcitonin level Ureteral dilatation in an ultrasound A predictive model designed to identify children (aged 1 month–4 years) presenting with a first febrile urinary tract infection (UTI) who are at risk of grade 3 or higher vesicoureteral reflux (VUR) to guide decisions about performing cystography.	Simple and easy to apply in clinical settings Requires minimal specialist information (only serum procalcitonin and ultrasound findings)	Moderate specificity (46%) leading to false positives Sensitivity decreased to 64% in the validation cohort Misdiagnosed 34% of children with high-grade VUR in a validation study Potentially affected by the timing of procalcitonin measurements	Internal	26
Lertdumrongluk et al., 2021 [46]	Lertdumrongluk Score	Variables included: Age > 6 months at presentation White blood cell (WBC) count ≥ 15,000/mm^3^ Presence of sepsis Abnormal renal ultrasound findings A scoring system developed from a retrospective study of 260 children (<72 months) with their first febrile urinary tract infection (UTI), who underwent renal ultrasound and voiding cystourethrography (VCUG) The score assigns points for each predictive variable, and the total score stratifies patients into low- and high-risk groups for vesicoureteral reflux (VUR), aiding the decision to perform VCUG.	Relies on simple, routinely available clinical and laboratory parameters Easy to calculate and apply in clinical settings Provides a practical risk stratification approach	Derived from a retrospective single-cohort analysis The reported predictive accuracy is moderate (70%)	None	13
Kurokawa et al., 2022 [47]	Kurokawa Score	Variables included: Age Sex Prolonged fever Hypoproteinaemia Hyponatraemia Hyperglycaemia A predictive scoring system developed in children under 2 years old with a first febrile urinary tract infection (UTI) caused by *E. coli* Derived from retrospective data collected at a single large teaching hospital in Japan, using two cohorts: A non-selective cohort (2007–2014): All the febrile UTI patients underwent VCUG. A selective cohort (2016–2019): Only high-risk patients underwent VCUG. The score identifies clinical and laboratory factors predictive of vesicoureteral reflux (VUR) and was shown to outperform ultrasound findings for detecting higher-grade VUR.	Uses simple, readily available clinical and biochemical data Can assist in reducing unnecessary VCUG procedures and radiation exposure Potentially applicable for early stratification of at-risk patients	A retrospective, single-centre study	None	0
Laleoğlu et al., 2025 [3]	A prediction model for high-grade VUR	Variables included: Age < 2 years Male sex Non-E. coli uropathogen Hydronephrosis classified as UTD-P3 urinary tract dilatation in an ultrasound (2 points) Multiple kidney scars on DMSA scintigraphy (2 points) Developed from a retrospective analysis of 1044 children who underwent VCUG due to UTI or dilated urinary tract in an ultrasound Identifies children at high risk for severe VUR (grade 4 or higher) using a point-based scoring system (range: 0–7) Risk categories: Low risk: 0–2 points Moderate risk: 3–4 points High risk: 5–7 points A score of 5 was found to have 50% sensitivity, 97.1% specificity, and 98.2% negative predictive value for predicting severe VUR.	Uses objective, quantifiable clinical and imaging parameters Provides clear stratification for decision making regarding VCUG necessity Demonstrates high specificity and negative predictive value Based on a relatively large dataset	Retrospective study design Relies on specialised imaging (ultrasound and DMSA scintigraphy), which may limit universal applicability Moderate sensitivity (50%)—risk of missed cases	Internal	0
Acronym Key						
Acronym AUC BBD CAP CRP DMSA *E. coli* GFR MAG3 NNT PUD ROC RIVUR UDR UTD-P3 UTI VCUG VUR VURx/VUR Index WBC	Full Term Area Under the Curve Bladder and Bowel Dysfunction Continuous Antibiotic Prophylaxis C-Reactive Protein Dimercaptosuccinic Acid (scan) Escherichia coli Glomerular Filtration Rate Mercaptoacetyltriglycine (scan) Number Needed to Treat Periureteral Diverticulum Receiver Operating Characteristic (curve) Randomised Intervention for Vesicoureteral Reflux (trial) Ureteral Diameter Ratio Urinary Tract Dilation—Postnatal Grade 3 Urinary Tract Infection Voiding Cystourethrogram Vesicoureteral Reflux Vesicoureteral Reflux Index White Blood Cell (count)		Description/Context A measure of model accuracy or discrimination (from ROC analysis). A functional disorder affecting bladder and bowel control. A preventive long-term antibiotic therapy for recurrent urinary tract infections. A serum marker for inflammation, often used to detect/monitor infections. A nuclear medicine scan used to assess renal cortical scarring or function. A common bacterial cause of urinary tract infections. A measure of kidney function. A renal scintigraphy used to assess renal drainage and function. The number of patients who need to receive a treatment to prevent one additional bad outcome. A congenital or acquired outpouching near the ureter. A graphical plot used to assess the diagnostic accuracy. A multicentre clinical trial evaluating antibiotic prophylaxis in cases of vesicoureteral reflux. The radiographic ratio used to quantify ureteral dilation in vesicoureteral reflux. The highest grade of postnatal urinary tract dilation classification (severe hydronephrosis). An infection involving the urinary system (bladder, ureters, and kidneys). An imaging study used to diagnose and grade vesicoureteral reflux. The backflow of urine from the bladder to the ureters/kidneys. A composite scoring tool for predicting reflux resolution in children. A laboratory measure of immune cell levels, often elevated in infection.			

### 7.2. The Leroy Clinical Decision Rule—Leroy et al. (2012) [45]

Leroy et al. (2012) derived a clinical decision rule to predict which children presenting with a first febrile urinary tract infection will go on to be diagnosed with grade 3 or higher vesicoureteral reflux and, therefore, would benefit from cystography [45].

The authors performed a reanalysis of data from eight institutions, gathered from previously published prospective cohort studies. They considered all the children from 1 month to 4 years old who presented with a first febrile UTI to hospital; 494 children were included. Variables assessed included the serum procalcitonin level, abnormal ultrasound findings (ureteral dilatation, pelvicalyceal dilatation, and renal length), age, sex, family history of uropathy, and serum C-reactive protein level at hospitalisation [45].

The procalcitonin and CRP levels, pelvicalyceal dilatation, and ureteral dilatation were all statistically significant for high-grade VURs on univariate analysis. All these variables were then entered into a predictive model, and a stepwise reduction procedure was performed, which revealed that only the serum procalcitonin level and ureteral dilatation in an ultrasound scan remained significantly associated with VURs of grade 3 or higher and contributed to the prediction according to a maximum likelihood ratio estimate. The final fit of the model was good, with a *p*-value of 0.2, and its area under the ROC curve being 0.75 [45].

The final rule recommended that cystography should be performed in cases with a serum procalcitonin level of ≥0.17 ng/mL and ureteral dilation in an ultrasound scan or without ureteral dilatation when the serum procalcitonin level is ≥0.63 ng/mL. The publishing team found that the rule yielded 86% sensitivity with 46% specificity [45].

The clinical rule was validated by the original publishing team, soon after the e-publication of the original manuscript, using a separate cohort of 413 children. They reported a specificity of 46%, unchanged from that in the original publication; however, the sensitivity dropped to 64%. This difference was speculated to be related to the timing of the procalcitonin level evaluation. In this cohort, 34% of the patients with high-grade VURs were misdiagnosed using the rule [48].

This clinical decision rule is simple to apply and requires minimal specialist information, making it an attractive option for clinical practice; however, currently, it has not been demonstrated in external validation studies to be reliable and, therefore, is not appropriate for use in general clinical practice.

### 7.3. The Lertdumrongluk Score—Lertdumrongluk et al. (2021) [46]

The Lertdumrongluk score was developed after a retrospective analysis of 260 children under 72 months of age who presented to a tertiary hospital in Thailand between January 2008 and December 2019 with a first febrile urinary tract infection and underwent renal ultrasound scanning and voiding cystourethrography during their admission. The authors performed a multivariate logistic regression analysis to identify variables that were significantly associated with an ongoing diagnosis of vesicoureteral reflux and concluded that the following factors were significant: an age of >6 months at presentation, a white blood cell count of greater than or equal to 15,000/mm^3^, the presence of sepsis, and abnormal renal ultrasound findings [46].

Lertdumrongluk et al. assigned points for the presence of each variable and used the total summed score to develop a binary scoring system: Patients with a score of 0–2 were stratified as low risk, and those with a score of >2 were stratified as high risk. By categorising the patients with a score of >2 as high risk and using this to facilitate the decision to perform a VCUG to diagnose VUR, Lertdumrongluk et al. were able to reduce the number of patients undergoing unnecessary VCUG, with a reported predictive accuracy of 70% [46].

The main advantages of the Lertdumrongluk score are that it relies on simple and readily available information early in the clinical course and is easy to calculate. However, to date, no external validation has been published, and, therefore, the score is not currently applicable outside of its derivation cohort.

### 7.4. The Kurokawa Predictive Score—Kurokawa et al. (2022) [47]

Kurokawa et al. (2022) [47] developed a predictive scoring system to attempt to improve the stratification of patients under 2 years of age presenting with a first febrile urinary tract infection secondary to an *E. coli* infection. Data were collected retrospectively regarding all the children who presented with a first febrile *E. coli* UTI within two distinct timeframes: January 2007–March 2014 and January 2016–December 2019. All the patients were treated at a single large teaching hospital in Japan. The specific timeframes given were chosen due to a policy change, whereby between 2007 and 2014, every patient presenting with a first febrile UTI underwent a VCUG, whereas between 2014 and 2019, only patients who had abnormal renal ultrasound findings, complications of bacteraemia, a non-E-coli-induced febrile UTI, acute focal bacterial nephritis or underwent a VCUG. Kurokawa thus dubbed the 2007–2014 cohort the ‘non-selective’ group and the 2014–2019 group the ‘selective’ cohort. The non-selective cohort consisted of 111 patients, and the selective cohort consisted of 102 patients [47].

The authors evaluated a variety of clinical and laboratory factors in the non-selective cohort and performed a multivariate analysis to identify factors that might discriminate which patients are likely to be diagnosed with VUR. They utilised the factors that they identified as being significantly predictive of diagnosing VUR and formulated a predictive score. They then applied the score to patients from the selective and non-selective cohorts and refined the predictive factors based on their results. The final score consisted of an age of <5 months at presentation (1 point), the female sex (2 points), a duration of fever of >3 days (1 point), a serum total protein level of <6.6 g/dL (2 points), a serum sodium level of <136 mEq/L (1 point), and a serum glucose level of >100 mg/dL (2 points). The total available points, therefore, is 9, and a value of ≥5 had 80.7% sensitivity, 62.9% specificity, a positive predictive value of 49.0%, and a negative predictive value of 88.0%. The overall area under the ROC curve was 0.8 [47].

The Kurokawa predictive score is a simple tool that utilises data that are readily available in patients being investigated for a febrile urinary tract infection. This makes it a promising way to stratify who needs to undergo further investigations with a dose of radiation. The primary limitation at this stage is that no external validation has been published, making it difficult to confidently say that the score is useful outside of its original cohort.

### 7.5. The Prediction Model for High-Grade VURs—Laleoğlu et al. (2025) [3]

Laleoğlu et al. (2025) [3] sought to improve the predictions of which children presenting a urinary tract infection and/or hydronephrosis to strategise who is likely to have an underlying diagnosis of severe vesicoureteral reflux (a VUR grade of 4 or greater) and, thus, would benefit from a voiding cystourethrogram [3].

The authors performed a retrospective analysis of 1044 patients who underwent a VCUG due to a urinary tract infection or a dilated urinary tract in ultrasonography and developed a predictive model using variables that they determined were significantly associated (*p* < 0.05) with VUR grades of 4 or above. The variables were selected using the chi-squared test. The odds ratio for each chosen variable was determined and divided by the lowest odds ratio to simplify the score. Cutoff values for each variable were then established for evaluating the sensitivity and specificity values and Youden’s Index. The variables chosen for inclusion in the model included an age of <2 years, the male sex, a non-E. coli uropathogen, hydronephrosis classified as UTD-P3 urinary tract dilatation in an ultrasound, and multiple kidney scars on DMSA scintigraphy, with 1 point assigned for the presence of all the variables except for hydronephrosis in an ultrasound and multiple kidney scars, which were assigned 2 points when present [3].

The total score, therefore, was between 0 and 7 points, and the authors classified patients as low risk if they scored 0–2 points, moderate risk if they scored 3–4 points, and high risk if they scored 5–7 points. The rate of severe VURs among children with a score of 5 was 37.5%, while it was only 1.8% in children with a score  of ≤4 (*p*  <  0.001). The sensitivity, specificity, PPV, NPV, and OR for a score of 5 for predicting severe VURs were 50.0%, 97.1%, 37.5%, 98.2%, and 33.6, respectively [3].

This prediction model shows promise for being a simple scoring system using variables that are generally easy to obtain, although ultrasound scanning and DSMA scanning do require a level of specialist investigation that might not be universally available. To date, no external validation of this predictive model has been published, and this will be necessary to demonstrate its widespread validity.

## 8. Discussion

Vesicoureteral reflux remains a clinical challenge both to streamline exposure to diagnostic modalities and to predict short- and long-term outcomes. Numerous scoring systems, nomograms, predictive tools/models, ratios, and computational models have been developed over the past four decades to aid clinicians in predicting which patients should be investigated for potential reflux and of those with a confirmed diagnosis, who is likely to experience spontaneous resolution, persistent disease requiring surgical intervention, or recurrent infections. The persistence in publications suggests that there remains uncertainty surrounding the best method to predict the disease progression and treatment response. To the best of our knowledge, this atlas serves as the first document to provide a comprehensive summary of current tools that have been developed to predict outcomes for patients with suspected or confirmed vesicoureteral reflux.

The predictive tools described in the literature demonstrate of a range of methodologies—from early anatomical or radiographic ratios to advanced machine-learning and computational models. This progression demonstrates not only the evolving evidence regarding individual predictive factors but also the technological advancements that have become available to individualise prognostic assessments. Whilst early assessments tended to focus on radiological parameters that can be reproduced with relative reliability to provide categorical risk stratification, more modern multifactorial indices and computer-based models often use a wide range of radiological, clinical, and biochemical data points to produce individualised numerical scores, which can be applied either as they are or then categorised into risk groups. Recent tools have leaned heavily into automated data processing and pattern recognition to enhance the predictive accuracy. Despite the relatively large number of predictive tools that have recently been published, none has demonstrated sufficient reliability, accessibility, and generalisability to achieve widespread clinical use in paediatric urology.

Several limitations consistently recur across the available tools: Most notably, the fact that most tools were derived using relatively small, single-centre retrospective datasets often collected over extensive time periods. This introduces challenges both with the acquisition of data for analysis, such as missing data points and variability in the methods used to initially gather, store, and present data points, and changes in diagnostic standards and management strategies over time. In combination, the heterogeneity in imagining practices/interpretation, reflux grading, and treatment standards makes it difficult to validate and compare different tools for clinical use. Even tools such as the vesicoureteral reflux index and nomograms published by Estrada et al. (2009) [25], which were developed using relatively large datasets, remain constrained by a lack of prospective external validation and inherent variability in the interpretation of voiding cystourethrograms.

Another significant challenge is the heterogeneity in inclusion criteria, outcome measurements, and statistical analyses, even when comparing tools with similar outcomes. Alongside the issue of small datasets and challenges in extracting meaningful information, this makes meta-analytic synthesis and the formation of universal recommendations or cutoff values challenging.

The transition towards computational and machine-learning models represents a promising new era of prognostic modelling. Several of these methods have demonstrated superior discriminatory ability when compared to those described in earlier publications of regression-based tools. However, their reliance on detailed and complete datasets containing diverse clinical information, complex computational capabilities, and imaging data with potential heterogeneity in interpretation and availability among patients and clinical settings serves as a current barrier to widespread real-world application.

Despite the variability in the designs, methodologies, and defined outcomes across the predictive tools described herein, several factors have consistently demonstrated prognostic value. The ureteral morphology, reflux grade, bladder dysfunction, and presence of renal scarring have been widely found to have consistent prognostic significance in patients with confirmed or suspected vesicoureteral reflux, suggesting that these may prove to be valuable focal points for the future development of predictive tools.

Ultimately, the field remains limited by a lack of multicentre, prospective validations of many of these predictive tools. Collaborative studies designed to evaluate and compare existing tools using uniform definitions of predictors and outcomes are essential. Incorporating advanced statistical methods and artificial intelligence technologies offers an opportunity to compare and combine currently published methods to develop a universal, evidence-based risk stratification model that can be used to guide both diagnostic decision making and management strategies in patients with vesicoureteral reflux. This will require a balance of technological sophistication and clinical practicality to ensure that the predictive tool is a useful adjunct to clinical practice rather than complicating everyday clinical work.

As this is an atlas-style narrative review, we did not undertake formal systematic review processes (e.g., PRISMA flow reporting, duplicate independent screening, or a structured risk-of-bias appraisal). Consequently, the possibility of selection bias in the tools identified and reporting bias within included studies cannot be excluded; we, therefore, emphasise validation status and generalisability when appraising each tool.

## 9. Conclusions

This atlas provides the first consolidated overview of the current predictive tools designed to aid clinicians’ prognostications when considering investigative and treatment pathways for paediatric patients with suspected or confirmed vesicoureteral reflux. Across 17 described tools, ranging from traditional anatomical ratios to modern machine-learning models, several consistent goals emerge: to improve risk stratification, reduce unnecessary invasive or potentially harmful investigations, and provide individualised treatment plans to optimise patient care. However, despite decades of research and progress, no predictive tool has been demonstrated to be universally applicable. The principal limitations are the reliance on retrospective, single-centre datasets, variability in definitions, and a lack of prospective validation. The convergence of simple clinical predictors and advanced computational modelling represents a promising direction; however, rigorous multicentre validation across a diverse population will be required to translate these advances to practical clinical tools.

We suggest, therefore, that future work includes developing a standardised, multicentre, and prospectively validated predictive framework that incorporates established clinical variables and the analytical power of artificial intelligence. Such a model could provide the necessary transparency, accessibility, and precision to achieve universal adoption—transforming risk evaluation and personalisation of care for patients with vesicoureteral reflux.

## Data Availability

No new data were created or analysed in this study. Data sharing is not applicable to this article.

## References

[1] Puri P., Gosemann J.H., Darlow J., Barton D.E. (2011). Genetics of vesicoureteral reflux. Nat. Rev. Urol..

[2] Greenbaum L.A., Mesrobian H.G. (2006). Vesicoureteral reflux. Pediatr. Clin. N. Am..

[3] Laleoğlu P., Yildiz G., Bayram M.T., Uçar H.G., Kavukcu S., Soylu A. (2025). Prediction model for severe vesicoureteral reflux in children with urinary tract infection and/or hydronephrosis. Pediatr. Nephrol..

[4] Craig J.C., Irwig L.M., Knight J.F., Roy L.P. (2000). Does treatment of vesicoureteric reflux in childhood prevent end-stage renal disease attributable to reflux nephropathy?. Pediatrics.

[5] Ming J.M., Lee L.C., Chua M.E., Zhu J., Braga L.H., Koyle M.A., Lorenzo A.J. (2019). Population-based trend analysis of voiding cystourethrogram ordering practices in a single-payer healthcare system before and after the release of evaluation guidelines. J. Pediatr. Urol..

[6] Chirico V., Tripodi F., Lacquaniti A., Monardo P., Conti G., Ascenti G., Chimenz R. (2023). Therapeutic Management of Children with Vesicoureteral Reflux. J. Clin. Med..

[7] Tullus K., Shaikh N. (2020). Urinary tract infections in children. Lancet.

[8] Yazılıtaş F., Özlü S.G., Aydoğ Ö., Bülbül M., Çakıcı E.K., Karacan C.D., Yılmaz E., Çınar H.G., Şenel S. (2025). Voiding cystourethrography practices: Experiences in a tertiary pediatric referral hospital. Acta Radiol..

[9] Herndon A., Otero H.J., Hains D., Sweeney R.M., Lockwood G.M., Cost N., Canon S., Dean G., Kaefer M., Kieran K. (2025). Perinatal Urinary Tract Dilation: Recommendations on Pre-/Postnatal Imaging, Prophylactic Antibiotics, and Follow-up: Clinical Report. Pediatrics.

[10] Arant B.S. (1992). Medical management of mild and moderate vesicoureteral reflux: Followup studies of infants and young children. A preliminary report of the Southwest Pediatric Nephrology Study Group. J. Urol..

[11] Silva J.M., Santos Diniz J.S., Marino V.S., Lima E.M., Cardoso L.S., Vasconcelos M.A., Oliveira E.A. (2006). Clinical course of 735 children and adolescents with primary vesicoureteral reflux. Pediatr. Nephrol..

[12] Williams G., Fletcher J.T., Alexander S.I., Craig J.C. (2008). Vesicoureteral reflux. J. Am. Soc. Nephrol..

[13] Garcia-Roig M.L., Kirsch A.J. (2016). Urinary tract infection in the setting of vesicoureteral reflux. F1000Res.

[14] Mattoo T.K., Mohammad D. (2022). Primary Vesicoureteral Reflux and Renal Scarring. Pediatr. Clin. N. Am..

[15] Hunziker M., Colhoun E., Puri P. (2013). Prevalence and predictors of renal functional abnormalities of high grade vesicoureteral reflux. J. Urol..

[16] Hellström M., Hjälmås K., Jacobsson B., Jodal U. (1986). Ureteral diameter in low-risk vesicoureteral reflux in infancy and childhood. Acta Radiol. Diagn..

[17] Cooper C.S., Birusingh K.K., Austin J.C., Knudson M.J., Brophy P.D. (2012). Distal ureteral diameter measurement objectively predicts vesicoureteral reflux outcome. J. Pediatr. Urol..

[18] Arlen A.M., Kirsch A.J., Leong T., Cooper C.S. (2017). Validation of the ureteral diameter ratio for predicting early spontaneous resolution of primary vesicoureteral reflux. J. Pediatr. Urol..

[19] Arlen A.M., Leong T., Joseph Guidos P., Alexander S.E., Cooper C.S. (2017). Distal Ureteral Diameter Ratio is Predictive of Breakthrough Febrile Urinary Tract Infection. J. Urol..

[20] Wong S.M., Tseng C.-S., Hong J.-H., Huang K.-H., Chiang I.-N. (2023). Cutoff Value of Ureteral Diameter Ratio for Predicting Spontaneous Resolution of Vesicoureteral Reflux. Urol. Sci..

[21] Krishnan N., Agarwal P., Verma A., Sharma S., Yadav D.K., Kandasamy D., Anand S. (2024). Utility of ureteral diameter ratio for clinical decision-making in children with vesicoureteral reflux: A systematic review and meta analysis. Pediatr. Surg. Int..

[22] Cooper C.S., Orzel J.A., Bonnett M.A., Zimmerman M.B., Malicoat J.R., Hlas A.C., Storm D.W., Lockwood G.M. (2024). The impact of the distal ureteral diameter ratio, bladder volume at onset of vesicoureteral reflux, and/or grade in predictive models of clinical outcomes in children with vesicoureteral reflux. J. Pediatr. Urol..

[23] Cooper C.S., Orzel J., Bonnett M.A., Malicoat J.R., Hlas A.C., Lockwood G.M., Bridget Zimmerman M. (2025). The significant impact of the distal ureteral diameter ratio as predictor of breakthrough urinary tract infections in children with vesicoureteral reflux. J. Pediatr. Urol..

[24] Kirsch A.J., Arlen A.M., Leong T., Merriman L.S., Herrel L.A., Scherz H.C., Smith E.A., Srinivasan A.K. (2014). Vesicoureteral reflux index (VURx): A novel tool to predict primary reflux improvement and resolution in children less than 2 years of age. J. Pediatr. Urol..

[25] Estrada C.R., Passerotti C.C., Graham D.A., Peters C.A., Bauer S.B., Diamond D.A., Cilento B.G., Borer J.G., Cendron M., Nelson C.P. (2009). Nomograms for predicting annual resolution rate of primary vesicoureteral reflux: Results from 2462 children. J. Urol..

[26] Sjöström S., Pivodic A., Abrahamsson K., Sixt R., Stokland E., Hansson S. (2020). A scoring system for predicting downgrading and resolution of high-grade infant vesicoureteral reflux. Acta Paediatr..

[27] Knudson M.J., Austin J.C., Wald M., Makhlouf A.A., Niederberger C.S., Cooper C.S. (2007). Computational model for predicting the chance of early resolution in children with vesicoureteral reflux. J. Urol..

[28] Nepple K.G., Knudson M.J., Austin J.C., Wald M., Makhlouf A.A., Niederberger C.S., Cooper C.S. (2008). Adding renal scan data improves the accuracy of a computational model to predict vesicoureteral reflux resolution. J. Urol..

[29] Tafazoli N., Kamran H., Bazargani R., Samaei M., Naseri M., Kajbafzadeh A.M. (2025). Machine learning-based prediction of vesicoureteral reflux outcomes in infants under antibiotic prophylaxis. Sci. Rep..

[30] Arlen A.M., Garcia-Roig M., Weiss A.D., Leong T., Cooper C.S., Kirsch A.J. (2016). Vesicoureteral Reflux Index: 2-Institution Analysis and Validation. J. Urol..

[31] Garcia-Roig M., Ridley D.E., McCracken C., Arlen A.M., Cooper C.S., Kirsch A.J. (2017). Vesicoureteral Reflux Index: Predicting Primary Vesicoureteral Reflux Resolution in Children Diagnosed after Age 24 Months. J. Urol..

[32] Arlen A.M., Leong T., Wu C.Q., Traore E.J., Cooper C.S., Kirsch A.J. (2020). Predicting Breakthrough Urinary Tract Infection: Comparative Analysis of Vesicoureteral Reflux Index, Reflux Grade and Ureteral Diameter Ratio. J. Urol..

[33] Shiraishi K., Matsuyama H., Nepple K.G., Wald M., Niederberger C.S., Austin C.J., Cooper C.S. (2009). Validation of a prognostic calculator for prediction of early vesicoureteral reflux resolution in children. J. Urol..

[34] Dias C.S., Silva J.M.P., Diniz J.S.S., Lima E.M., Marciano R.C., Lana L.G., Trivelato A.L.L., Lima M.S., e Silva A.C.S., Oliveira E.A. (2010). Risk factors for recurrent urinary tract infections in a cohort of patients with primary vesicoureteral reflux. Pediatr. Infect. Dis. J..

[35] Hidas G., Billimek J., Nam A., Soltani T., Kelly M.S., Selby B., Dorgalli C., Wehbi E., McAleer I., McLorie G. (2015). Predicting the risk of breakthrough urinary tract infections: Primary vesicoureteral reflux. J. Urol..

[36] Yang Z., Li J., Liu P., Sun N., Song H., Zhang W. (2025). Development of a prediction model for risk of breakthrough urinary tract infection in children with primary vesicoureteral reflux. Transl. Androl. Urol..

[37] Troesch V.L., Wald M., Bonnett M.A., Storm D.W., Lockwood G.M., Cooper C.S. (2021). The additive impact of the distal ureteral diameter ratio in predicting early breakthrough urinary tract infections in children with vesicoureteral reflux. J. Pediatr. Urol..

[38] Wang Z.T., Wehbi E., Alam Y., Khoury A. (2018). A Reanalysis of the RIVUR Trial Using a Risk Classification System. J. Urol..

[39] Mattoo T.K., Carpenter M.A., Moxey-Mims M., Chesney R.W. (2015). The RIVUR trial: A factual interpretation of our data. Pediatr. Nephrol..

[40] Bertsimas D., Li M., Estrada C., Nelson C., Wang H.-H.S. (2021). Selecting Children with Vesicoureteral Reflux Who are Most Likely to Benefit from Antibiotic Prophylaxis: Application of Machine Learning to RIVUR. J. Urol..

[41] Oostenbrink R., van der Heijden A.J., Moons K.G., Moll H.A. (2000). Prediction of vesico-ureteric reflux in childhood urinary tract infection: A multivariate approach. Acta Paediatr..

[42] Leroy S. (2006). Prediction of vesicoureteral reflux after a first febrile urinary tract infection in children: Validation of a clinical decision rule. Arch. Dis. Child..

[43] Sánchez Bayle M., Yep Chullen G., de La Torre Montes de Neira E., Cano Fernández J., Rabadán Sanz B. (2008). Can vesicoureteral reflux be predicted in infants with urinary infection?. Nefrologia.

[44] Venhola M., Huttunen N.-P., Renko M., Pokka T., Uhari M. (2010). Practice Guidelines for Imaging Studies in Children After the First Urinary Tract Infection. J. Urol..

[45] Leroy S., Romanello C., Smolkin V., Galetto-Lacour A., Korczowski B., Tuerlinckx D., Rodrigo C., Gajdos V., Moulin F., Pecile P. (2012). Prediction of moderate and high grade vesicoureteral reflux after a first febrile urinary tract infection in children: Construction and internal validation of a clinical decision rule. J. Urol..

[46] Lertdumrongluk K., Lertdumrongluk P. (2021). Predictive score for vesicoureteral reflux in children with a first febrile urinary tract infection. Int. J. Urol..

[47] Kurokawa M., Murata K., Hoshina T., Furuno K., Kaku Y., Kishimoto J., Ohga S. (2022). A predictive score for detecting vesicoureteral reflux in children with their first Escherichia coli-induced urinary tract infection. Int. J. Urol..

[48] Leroy S., Bouissou F., Fernandez-Lopez A., Gurgoze M.K., Karavanaki K., Ulinski T., Bressan S., Vaos G., Leblond P., Coulais Y. (2011). Prediction of high-grade vesicoureteral reflux after pediatric urinary tract infection: External validation study of procalcitonin-based decision rule. PLoS ONE.

